# Evidence synthesis and decision modelling to support complex decisions: stockpiling neuraminidase inhibitors for pandemic influenza usage

**DOI:** 10.12688/f1000research.9414.2

**Published:** 2017-03-16

**Authors:** Samuel I. Watson, Yen-Fu Chen, Jonathan S. Nguyen-Van-Tam, Puja R. Myles, Sudhir Venkatesan, Maria Zambon, Olalekan Uthman, Peter J. Chilton, Richard J. Lilford

**Affiliations:** 1 Warwick Medical School, University of Warwick, Coventry, CV4 7AL, UK; 2 School of Medicine, University of Nottingham, Nottingham, NG7 2UH, UK; 3 Public Health England, London, SE1 8UG, UK; 4 Warwick Business School, University of Warwick, Coventry, CV47AL, UK

**Keywords:** Pandemic influenza, evidence synthesis, bias modelling, neuraminidase inhibitors, stockpiling

## Abstract

**Objectives: **The stockpiling of neuraminidase inhibitor (NAI) antivirals as a defence against pandemic influenza is a significant public health policy decision that must be made despite a lack of conclusive evidence from randomised controlled trials regarding the effectiveness of NAIs on important clinical end points such as mortality. The objective of this study was to determine whether NAIs should be stockpiled for treatment of pandemic influenza on the basis of current evidence.

**Methods**: A decision model for stockpiling was designed. Data on previous pandemic influenza epidemiology was combined with data on the effectiveness of NAIs in reducing mortality obtained from a recent individual participant meta-analysis using observational data. Evidence synthesis techniques and a bias modelling method for observational data were used to incorporate the evidence into the model. The stockpiling decision was modelled for adults (≥16 years old) and the United Kingdom was used as an example. The main outcome was the expected net benefits of stockpiling in monetary terms. Health benefits were estimated from deaths averted through stockpiling.

**Results**: After adjusting for biases in the estimated effectiveness of NAIs, the expected net benefit of stockpiling in the baseline analysis was £444 million, assuming a willingness to pay of £20,000/QALY ($31,000/QALY). The decision would therefore be to stockpile NAIs. There was a greater probability that the stockpile would not be utilised than utilised. However, the rare but catastrophic losses from a severe pandemic justified the decision to stockpile.

**Conclusions**: Taking into account the available epidemiological data and evidence of effectiveness of NAIs in reducing mortality, including potential biases, a decision maker should stockpile anti-influenza medication in keeping with the postulated decision rule.

## Introduction

Like many other potentially catastrophic events for which governments need to prepare, influenza pandemics are rare. Although the risk is considered to be 3–4% per annum ^1^, the public health consequences are widely recognised to be potentially severe ^2^. The epidemiology of only a small number of influenza pandemics has been well studied and evidence for the effectiveness of remedial influenza treatments in a pandemic scenario is scant. Yet, governments around the world still have to decide whether or not to stockpile anti-influenza medication like neuraminidase inhibitor (NAI) antivirals, such as oseltamivir (Tamiflu®) and zanamivir (Relenza®), as a defence against pandemic influenza.

The stockpiling of NAIs has been a controversial issue. Firstly, stockpiling may be seen to be a waste of large amounts of public money if the pandemic fails to materialise or if it is mild. In the United Kingdom, the previous Chief Medical Officer was criticised for spending £560 million on medicine that went largely unused in the 2009–10 pandemic ^3^. However, taking a default position of not stockpiling, or making the decision on the basis of intuition alone, is not justifiable given the rare but potentially catastrophic losses associated with pandemic influenza and the large cost of stockpiling.

Secondly, there has been a lack of conclusive evidence on the effectiveness of NAIs. Recent meta-analyses of randomised controlled trials (RCT) of seasonal influenza cases demonstrated reductions in rates of hospitalization, lower respiratory complications, and a decreased time to symptom alleviation but were unable to confirm or refute an effect of NAIs on more important clinical end points such as mortality ^4, 5^. A caveat of these studies, which were required for licensure of drug in healthy adults, is that they were not powered to determine low frequency but critical end points such as mortality in a largely healthy adult population. A further meta-analysis of observational data from pandemic influenza did find evidence of a reduction in the risk of mortality when NAIs were given to patients hospitalised with influenza ^6^. Some authors have criticised it for being subject to a large degree of bias and rejected it as a suitable form of evidence with which to formulate policy decisions ^7, 8^, though others argue that this evidence strongly supports the use of NAI treatment for influenza in hospitalised patients ^9^.

Evidence that has a bearing on death rates is not confined to measurement of mortality alone – there are other sources of relevant evidence. Clinical trials show that NAIs have beneficial effects on a number of outcomes as described above ^4– 6^. The treatment has a plausible rationale and it works *in vitro* and in animal models for this zoonosis ^10^. An arguably extreme position is to assume that these observations contain no information regarding effectiveness in preventing the rarer, but more severe outcomes, such as death. People who take to heart Bradford Hill’s list of factors that should affect the interpretation of data ( Box 1), would reject such a completely non-theoretical stance. But even within this framework conflicting conclusions may still be drawn, especially when inappropriately filtered through the lens of statistical significance. Estimation of potentially small effect sizes on rare endpoints is often characterised by uncertain and often conflicting evidence and many recent studies do conflict with those that support the effectiveness of NAIs ^11– 13^. Both an observed reduction and an increase in the risk of mortality are therefore potentially consistent with the aforementioned evidence. There is thus a compelling case for the synthesis of and extrapolation from various forms of evidence in order to examine the investment decision facing decision makers.

**Box 1.** Criteria proposed by Sir Austin Bradford Hill for evaluating causation and application of the criteria to relevant evidence for neuraminidase inhibitors

Criteria Strength Consistency Specificity Temporality Biological gradient Plausibility Coherence Experiment Analogy	Evidence for neuraminidase inhibitors Reasonably large effect (OR=0.81, 0.71 to 0.94) in reducing mortality in hospitalised patients in individual participant data meta-analysis of observational evidence ^6^ A previous meta-analysis of observational studies have also shown significant reduction in mortality ^38^. Whether reduction in mortality was mainly attributed to reduction in death related to influenza did not seem to have been investigated. Meta-analysis of individual participant trial data has shown that reduction in time to symptom relief, lower respiratory complications and hospitalisation occurred among influenza-infected patients but not among uninfected patients ^4, 5^. Early administration of the medication is associated with better clinical outcomes ^4– 6^, although the temporal relationship between changes in influenza viral shedding and clinical outcomes have not been well- established ^39^. Dose-response was observed in some of the animal studies ^40^. It is biologically plausible that a medication inhibiting the replication of a virus will reduce the seriousness of its effects Evidence for anti-viral activities of the medication is reasonably coherent between laboratory studies and clinical observations ^40^. Randomised controlled trials, while under-powered for outcomes such as death and hospitalisation, show reduction in the duration of illness for treatment and reduction in symptomatic influenza for prophylaxis ^4, 5^. Prophylactic antiviral medications that reduce cytomegalovirus infection also reduce associated death in organ transplant recipients ^41^.

Previous studies have estimated how cost-effective NAI stockpiling would be under a range of different pandemic influenza scenarios ^14– 19^. Stockpiling is generally estimated to be cost-effective. However, these studies took observational evidence of effectiveness, often from seasonal influenza studies, at face value and did not model potential biases that may have led to overestimation of benefits. Moreover, they only examined a limited number of specific future scenarios. The results of such cost-effectiveness models hinge on the available evidence of effectiveness and it may not be immediately clear to decision makers the implications of new evidence. We have therefore taken a different approach.

The calculation of the number of deaths from an influenza pandemic is simply calculated from a number of relevant variables such as the size of the population, the clinical attack rate, and the case fatality ratio. The effectiveness of NAIs in terms of relative risks can then be used to estimate the potential number of deaths averted through their use. A simple model can provide a useful framework to synthesise the available evidence while also remaining clear and transparent to decision makers. There is a large degree of uncertainty regarding the variables in the model, due to factors such as random mutations in the influenza virus, individual behaviour, and distribution of NAIs, nevertheless appropriate distributions can be specified for each variable and the uncertainty propagated through the model to estimate the distribution of possible numbers of deaths and resulting QALYs under the stockpiling and no stockpiling options. The model presented here exemplifies an approach to decision making under the types of uncertainty described above using a simple, transparent model to assist decision makers and to help inform the stockpiling decision.

## Methods

### Modelling approach

The methods used in this study are founded in normative decision theory ^20, 21^, which considers what decisions we ought to take, and Bayesian statistics. We used a well-established technique based on expected utility theory ^20, 21^ to model the binary decision to stockpile or not to stockpile NAIs. Within this framework, the decision simplifies to a question of whether the expected net benefits of the stockpiling decision are positive ^22^.

The net benefit associated with stockpiling was set as the value of the deaths averted minus the costs of stockpiling. If the expected net benefit of stockpiling is positive then the decision would be to stockpile, and if it is negative, not to stockpile.

The value of the deaths averted was modelled as:

                     Pop × Prob × CAR × CFR × Hospital × Treated × (1 – θ) × QALY × λ

**Figure 1.**
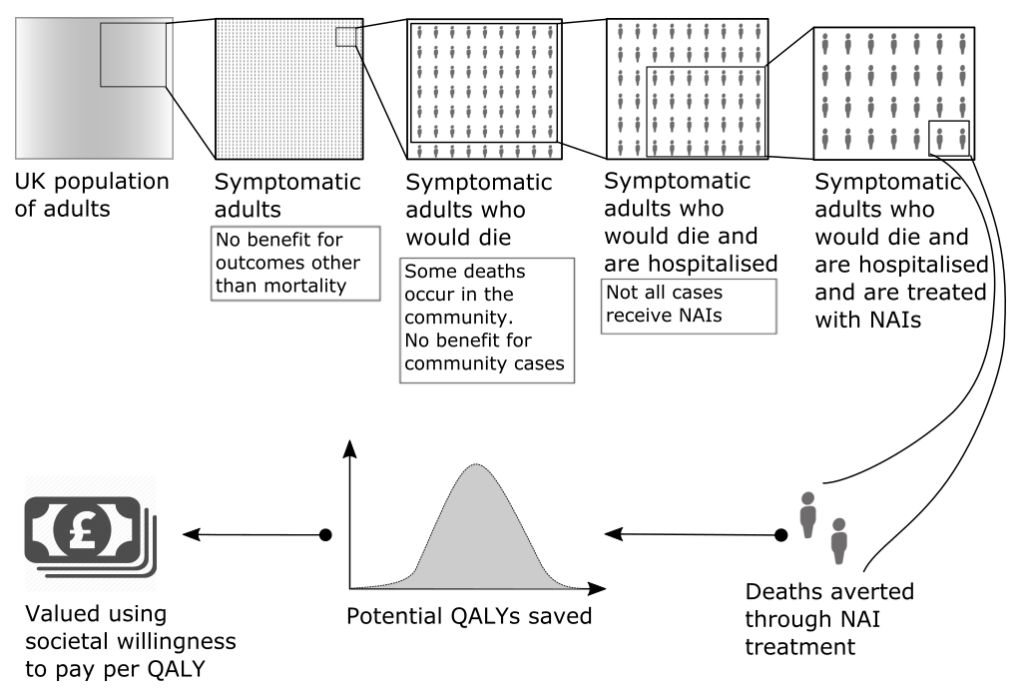
Explanation of the model used to derive the value of the deaths averted due to neuraminidase inhibitor stockpiling.

Firstly, the number of pandemic influenza deaths was calculated by multiplying the number of adults in the UK (Pop) by: the probability of there being a pandemic within the stockpile shelf-life (Prop), the clinical attack rate (CAR), and the case fatality ratio (CFR). We further multiply by the probability a pandemic influenza death occurred in hospital (Hospital), and the probability one of these patients receives NAIs (Treated). The number of deaths averted by NAI treatment in this population of NAI-treated adults was given by the relative risk reduction in mortality associated with NAI treatment (1 – θ). Finally, the value of these deaths averted was calculated by multiplying by the quality adjusted life years (QALY) associated with each pandemic influenza mortality (QALY), and the societal willingness to pay per QALY (λ). This model is further explicated in Figure 1.

We considered reductions in mortality among symptomatic adults resulting from stockpiling, but did not take into account possible additional effects on complications such as pneumonia or that community use might reduce complications, hospitalisation, or mortality. Only adults were considered on the grounds that NAI effectiveness ^4, 6^ is less certain in children and to determine if the decision to stockpile could be justified on the basis of any benefit among adults alone.

Decision modelling is founded in the Bayesian paradigm, which was used to evaluate the stockpiling decision for a future pandemic with unknown epidemiological variables and unknown effectiveness of NAI. A sub-model was specified for each epidemiological variable in the decision model. Data from previous pandemics were assumed to be observations from an underlying common distribution, the parameters of which were estimated using these data as described in the following section. The decision was then evaluated over posterior predictive distributions for the epidemiological parameters. We used a bias corrected effectiveness estimate for the effectiveness of NAIs as described below. The model was estimated using Markov Chain Monte Carlo (MCMC) with 10,000 iterations using R 3.2.3 and Stan 2.11.0. This method obviates the need to conduct separate probabilistic sensitivity analyses since the posterior distribution of the net benefits represents the uncertainty about future influenza pandemics and NAI effectiveness. The expected net benefits represent the gains or losses from stockpiling, on average, given the different distributions for the different parameters. Convergence of the MCMC chains was assessed by visual inspection of autocorrelation, running mean, and trace plots in R.

### Data and variables

The data and statistical code are provided with the paper.

### Influenza pandemic epidemiology

The data used to estimate the parameters in the model were obtained from documents compiled to assess pandemic influenza and thus represent the decision maker’s prior knowledge ^1^. The shelf-life of oseltamivir, the principle drug comprising the vast majority of the NAI stockpile, is ten years ^23^.

The clinical attack rate and case fatality ratios from previous pandemics were assumed to be observations from beta distributions. Improper non-informative priors with a lower limit of zero were assigned to the parameters of these distributions, which were then updated with the data from the previous pandemics. We excluded the observation of a clinical attack rate of 60% in the 1889–92 Asiatic flu pandemic as the UK government’s worst case scenario is a clinical attack rate of 50%. The probability that a pandemic occurs in the shelf life of the stockpile was similarly estimated from the data with each decade between 1900 and 2010 as a binary observation equal to one if a pandemic occurred in that decade and zero otherwise. These binary observations were assumed to be observations from a Bernoulli distribution.

### Effectiveness of neuraminidase inhibitors

No RCT evidence for the effectiveness of NAIs in reducing the risk of mortality in pandemic influenza was available. Too few deaths were observed in RCTs of seasonal influenza ^4^. We based our effectiveness estimate on a recently published pooled meta-analysis of observational, patient-level data from hospitalised pandemic influenza virus patients ^6^. We converted the odds ratios (OR) for mortality associated with NAIs (irrespective of time from onset) provided in the paper into relative risks (RR): *RR* = *OR*/(1 – *p* + ( *p* × *OR*)) where *p* is the baseline (approximately 10%) ^24^. The study was based on hospitalised patients, in order to apply the observed relative risk from hospitalised patients to the general population considered here, we made two conservative assumptions. First, we assumed that there would be no difference in the patients that would be hospitalised and those that would remain in the community in a no stockpile and stockpile scenarios. This is conservative because community treatment will be given earlier, on average, in the course of the disease if it can be administered in the community and there is evidence that the earlier the treatment is given, the better ^4– 6^. Secondly, we assume that only deaths occurring in hospital in the non-stockpile scenario would be averted under the counterfactual stockpile scenario. A study of mortality in the A/H1N1 2009 pandemic in England, found that 92% of deaths (125 of 136 cases studied) occurred in hospital ^25^. Assuming that none of these 8% of deaths taking place in a non-stockpile scenario would be averted under the counterfactual is as conservative as it can be. The logic of our approach is laid out in Figure 1.

### Bias modelling

In addition to these conservative assumptions regarding the application of in-hospital relative risk reductions to a community population, we also took into account the observational nature of the hospital based evidence itself. A number of authors have raised this issue in connection with the study used here ^7, 8^, although others dispute the strength of these criticisms ^9^. We used a method previously published elsewhere to model bias ^26^. Five reviewers (SIW, RJL, YFC, OU, and PJC) who were not associated with the observational data study independently completed a bias questionnaire and provided their beliefs about both additive and proportional bias present in the study across a range of domains. The reviewers were selected on the basis of their experience with observational data research and its associated biases, with expertise in health care and public health research. The median values for the mean and standard error of the bias across reviewers were used to ‘correct’ the observational evidence ^26^. The method for bias modelling used here was originally intended for individual studies so that they could be adjusted prior to an evidence synthesis ^26^. This method has been applied here since the study in question is an individual patient pooled meta-analysis, analysed using a similar method to that any single study would use, except that the data originate from multiple locations and are of varying quality. The reviewers considered this an additional source of uncertainty when evaluating the quality and potential for bias.

### QALY losses

The distribution for the average age associated with an influenza death in previous pandemics was assumed to be drawn from a scaled Beta distribution with an upper limit of 81.5, which is the UK life expectancy at birth. The parameters of this distribution were then estimated from data; the average ages of influenza deaths from prior pandemics were 27 (1918), 65 (1957), 62 (1968), and 45 (2009) ^25, 27, 28^, no data were available from the 1889–92 pandemic. To estimate QALYs lost due to an influenza death, the remaining life expectancy was calculated by differencing the average age at death from the UK life expectancy at birth (i.e. 81.5 years) ^24^. These years were weighted by the average QALY weight for a person aged over 45 of 0.8 ^25^, and then discounted at the rate of 3.5% per annum as recommended by the National Institute for Health and Care Excellence (NICE) ^29^.

### Other parameters

We also estimated the probability a pandemic influenza death occurred in hospital using data on 2009 pandemic influenza deaths ^25^. We further considered a number of scenarios for the distribution of NAIs and the proportion of symptomatic pandemic influenza cases that would receive the drug. Our base case was 100%, however we also considered the decisions that would be made in the range of 0% to 100% in a deterministic sensitivity analysis – the value of the deaths averted was multiplied by a number between zero and one. The cost of stockpiling was assumed fixed at £560 million ($860m, €750m) and was based on the figures quoted in the above mentioned Select Committee hearings ^3^. We considered the adult population of the UK, which was 50.5 million in 2015 ^30^. The willingness to pay per QALY was selected as £20,000/QALY ($31,000/QALY) for the base case analysis, the lower end of the range (£20,000-£30,000/QALY; $31,000–$45,000/QALY) specified by NICE as being cost-effective ^29^. We examined the decision that would be made under a range of willingness to pay per QALY values of £5,000/QALY ($7,500/QALY) to £30,000/QALY ($45,000/QALY).

## Results

### Summary of estimated parameters

Table 1 shows the posterior mean and 95% credible intervals for the parameters in the model. Using data from previous influenza pandemics, mean values (95% credible intervals) were as follows: clinical attack rate 23.8% (5.2%, 50.6%), case fatality ratio 0.7% (0.0%, 3.0%), and probability of experiencing a pandemic within a decade 38.5% (15.3%, 64.9%). The expected value for the mean QALY losses associated with influenza mortality was 15.2 (5.7, 20.9). The proportion of pandemic influenza deaths that occurred in hospitalised patients was 91.9% (86.9%, 95.8%).

**Table 1.** Summary of posterior distributions of the model parameters.

Parameter	Mean Value (95% Credible interval)	Source
Costs ^a^	£560,000,000	3
Willingness to pay per QALY	£20,000/QALY	29
Probability of pandemic in shelf life	38.7% (15.3%, 64.9%)	1, 23
Adult Population	50.5 million	30
CAR ^b^	23.3% (5.2%, 50.6%)	1
CFR ^b^	0.72% (0.01%, 2.97%)	1
QALY loss, mortality	15.2 (5.7, 21.0)	25, 27, 28, 42, 43
Proportion of pandemic influenza deaths in hospitalised patients	91.9% (86.9%, 95.8%)	25
Oseltamivir Effectiveness, mortality (relative risk)	0.83 ^c^ (0.71, 0.94)	6
Bias corrected Oseltamivir Estimate (relative risk)	0.89 (0.71, 1.07)	6, 26, Five independent assessors

CAR = clinical attack rate; CFR = case fatality ratio. Probabilities expressed as %. ^a^Assumed to be fixed. ^b^See Appendix A for derivation. ^c^Relative risks converted from odds ratios (0.81, 95% CI: 0.70, 0.93) using a baseline risk of mortality of 10% ^19^.

The observed relative risk was 0.83 (95% confidence interval: 0.71, 0.94) and the bias corrected relative risk was estimated as 0.89 (0.71, 1.07). The principle sources of bias identified by the reviewers were selection bias, due to a lack of randomisation, the possibility that studies with a positive finding may have been more likely to volunteer their data for the meta-analysis, and attrition bias. Not all reviewers were in agreement about the overall effects of bias, but the median response was that there was an overestimation of treatment benefit.

### Main results

**Figure 2.**
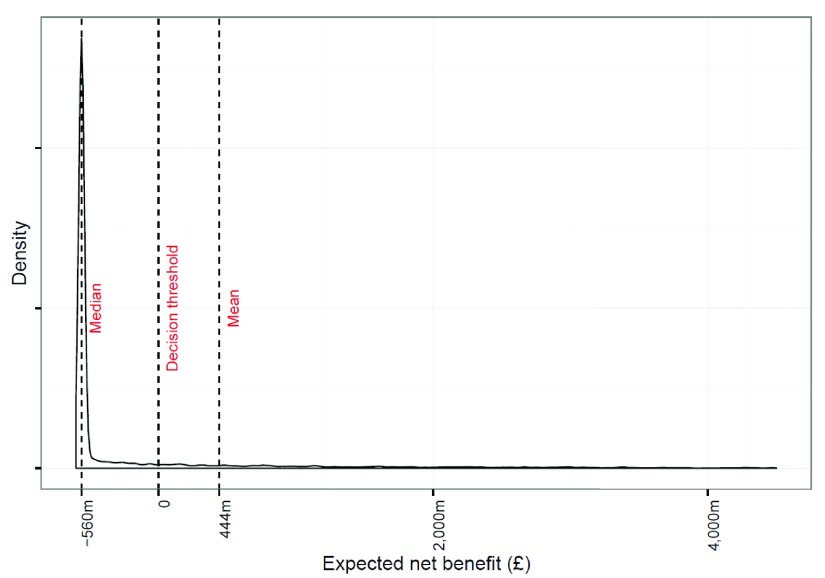
Posterior distribution of the loss function for stockpiling NAIs showing the mean and median values of the distribution along with the decision threshold for stockpiling. The x-axis has been truncated at £4·5b.

Table 2 shows the results from various scenarios considered. The expected net benefit of stockpiling in the baseline analysis was £444 million ($668 million). The decision would be therefore to stockpile NAIs. Figure 2 shows the posterior distribution of net benefits. The mean number of deaths averted was 3,218. There was a 77% probability that the benefits were negative implying that no pandemic occurred, an insufficiently large pandemic occurred, or NAIs were not effective enough to justify the stockpile. The median net benefit was £-560 million in each case as in the majority of scenarios no pandemic occurred and there was only the net cost of the stockpile. Nevertheless, the mean estimated net benefit was positive, which was caused by the very large number of deaths, many of which may be prevented by stockpiling, in the unlikely event of a severe pandemic. This can be seen in the long tail on the left of the distribution in Figure 2.

**Table 2.** Results from baseline analysis and secondary analysis varying the proportion of hospitalised cases receiving NAIs.

	Hospitalised patients with influenza receiving NAIs (%)	Expected net benefit (£m), (95% credible interval)	Median net benefit	Decision
1	100	444 (-808, 8,383)	-560	Stockpile
2	70	143 (-734, 5,700)	-560	Stockpile
3	50	-58 (-684, 3,911)	-560	Not Stockpile
4	30	-259 (-634, 2,123)	-560	Not Stockpile

The decision is to stockpile if the expected net benefit is greater than zero and not to stockpile otherwise. The willingness to pay per QALY is £20,000/QALY in all scenarios.

**Figure 3.**
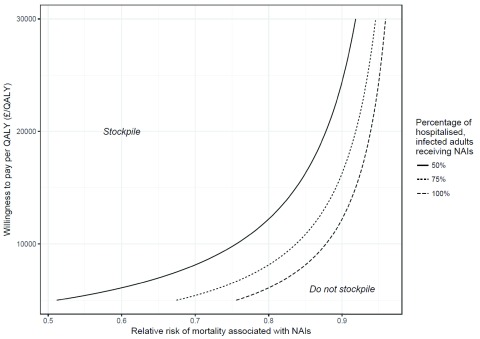
Stockpiling decisions that would be made under a range of different values for the effectiveness of NAIs, the percentage of hospitalised, symptomatic adults that would receive NAIs, and thresholds for willingness to pay per QALY. The lines represent thresholds for decision makers. For any point inside the region bounded by a given line the decision maker should stockpile and conversely any point outside that region the decision maker should not stockpile.

Figure 3 shows the decision under a range values for the effectiveness of NAIs, the percentage of hospitalised, symptomatic adults who would receive NAIs and willingness to pay per QALY threshold. If 100% of hospitalised, symptomatic adults with influenza received NAIs then the decision would be to stockpile as long as our threshold willingness to pay per QALY was greater than £11,116/QALY under our ‘bias corrected’ effectiveness estimate. When only 50% of hospitalised, symptomatic adults receive NAIs this threshold increases to £22,232/QALY, which would still be considered cost-effective in the range considered by NICE. The minimum percentage of hospitalised, symptomatic adults with influenza that would need to receive NAIs for the decision to be to stockpile at a threshold willingness to pay of £20,000/QALY is 56%. Conversely, when the proportions of hospitalised, symptomatic adults who receive NAIs is 50%, 75%, or 100%, the minimum value for the relative risk of mortality associated with NAIs required for the intervention to be considered cost-effective at a £20,000 per QALY threshold is 0.88, 0.92, and 0.94, respectively.

Raw data of ‘stockpiling neuraminidase inhibitors for pandemic influenza usage’ Click here for additional data file. Copyright: © 2017 Watson SI et al. 2017 Data associated with the article are available under the terms of the Creative Commons Zero "No rights reserved" data waiver (CC0 1.0 Public domain dedication).

## Discussion

This study has found that the available evidence suggests that stockpiling NAIs for pandemic influenza is rational under a range of assumptions. Many of these assumptions are conservative, such as no reduction in adverse clinical outcomes other than mortality, no benefit in patients who would not have been hospitalised had there been no stockpile, and no effect in children. However, this decision required at least 56% of the influenza patients who would have died without a stockpile to receive NAIs if the threshold willingness to pay was £20,000/QALY. In the 2009 pandemic, 64% of hospitalised patients received NAIs ^6^, and in the United Kingdom specifically this proportion was 75% ^31^, suggesting that 56% is achievable, and that therefore, stockpiling is supported by the available evidence.

This paper is predicated on the purchase of a stockpile large enough to treat a large proportion of the population (80% in the UK) in the community and in hospital with NAIs. This may well be the correct strategy if new evidence emerges that community-based treatment reduces either complications, hospitalisations or mortality. Further research will be required; indeed, the Bayesian decision analysis used here can be extended to consider how much to stockpile rather than simply whether to stockpile. However, if the evidence base were to remain limited to mortality reductions in hospitalised patients, or if the societal willingness to pay per QALY was low, as it may be in many resource poor settings, a ‘hospital-treatment only’ policy might be considered. This would reduce the cost of the stockpile significantly. For example, in the 2009 pandemic only 0.5% of symptomatic cases were hospitalised ^32^, these patients would require far fewer doses than the 1.16 million courses (at a minimum) of NAIs dispensed in the 2009 pandemic ^33^. For a population of 50.5 million adults with a CAR of 25%, a hospitalisation probability of 0.5% would lead to only approximately 60,000 admissions. The evidence also suggests that more timely treatment of NAIs (within two days of symptom onset) is more effective than treatment at any point ^6^, which would suggest that the effectiveness of NAIs could be more favourable than modelled under the stockpiling policy. In all cases the decision would remain to stockpile NAIs.

Our conclusions are in line with the decision that would be made on the basis of cost-effectiveness evidence from previous studies ^14– 19^. However, our study does not take observational evidence at face value, but ‘downgrades’ it, thereby yielding a reduced estimate of effectiveness and wider credible limits. We have calculated the distribution of possible deaths from pandemic influenza using a relatively simple mathematical model and then ‘averaged’ over the distributions of the variables rather than examining cost-effectiveness on a scenario-by-scenario basis. This approach is intuitively simple and is aimed to provide correct inferences using a simple logical framework for the synthesis of the commonly available evidence in order to assist decision makers with a complex decision. The model allows the logical basis of the decision to be ‘reverse engineered’, allowing the decision to be critiqued within the framework established by the model. We note though that many people are highly sceptical about the benefits of NAIs. This study is not intended to be proscriptive. As Figure 3 illustrates, a decision maker with a highly sceptical belief about NAI effectiveness should not stockpile.

Obtaining an estimate for the bias in any particular study, or consolidated group of studies, is clearly an uncertain undertaking. There is an evidence base on bias arising from meta-regressions or other analyses comparing the results of imperfect studies to those of a ‘gold standard’. A recent Cochrane review comparing treatment effects reported in observational studies as compared to RCTs found that, “on average, there is little evidence for significant effect estimate differences between observational studies and RCTs...” ^34^ It is not surprising, given the considerable uncertainties surrounding the meta-analysis cited here, that the differences between the reported effects and our bias corrected effect resembles the differences in empirical studies comparing observational studies and RCTs ^34– 36^.

We acknowledge weaknesses in our study. The only outcome considered in the analyses was mortality. Adverse events caused by NAIs may also generate increased costs and hence reduced benefit. For example, a review of clinical trial evidence of NAIs found an increased risk of nausea and vomiting associated with treatment ^4^. The authors also reported a possible increase in the risk of psychiatric adverse events. However, this only reached statistical significance in exploratory analyses including a supra-licence dose and off-treatment periods. A more recent meta-analysis based on individual-level patient data of clinical trials focusing on licensed dose only found no such effects, but the number of events was small ^5^. Neuraminidase inhibitors may also have protective effects against some adverse events such as cardiac events, and may reduce the risk of influenza-associated pneumonia and hospitalisation ^4, 5^. The benefit of treatment is unlikely to be grossly over-estimated and is likely to be under-estimated given our conservative assumptions. We have also not considered potential effects on children or from reductions in complications, hospitalisations or mortality that might be associated with community-based treatment, or any benefit arising from changing disease dynamics and reduced transmission; nor have we considered wider societal effects, such as productivity gains, reduced community transmission, and the value placed on a stockpile for a potentially risk averse population, all of which may increase the benefits of stockpiling.

We note that our analysis is focussed on the United Kingdom but that it may be of use to other countries. The model for the benefits of NAIs can be simply applied to new contexts. However, the determination of the costs of the stockpile remains difficult. The costs depend on the treatment strategy planned for a given country and any price negotiations between the manufacturer and the government. A useful tool in this context is the ‘headroom’ method that asks instead what the maximum amount a decision maker should be willing to pay for an intervention, given a willingness to pay per unit benefit. This is a useful direction for future research.

We have assumed independence between the clinical attack rate and case fatality ratio, as well as other variables, however there is some evidence to suggest that they could be correlated ^37^. Nevertheless, the data are admittedly scant, and it is expected that this is a neutral assumption. Of course, if they are positively correlated then our conclusions become more conservative.

Our model examines the decision in the abstract and does not concern itself with externalities such as the possibility that availability of the drug will affect attitudes and hinder the effort to contain the spread of the disease, or that resistance to antivirals may develop. Nor have we considered the sensitivity of clinical diagnosis of influenza in identifying true positives or the costs and logistics of establishing a distribution process for the NAIs. The propensity to consult is also an important factor that may have affect the proportion of true positives, which in turn may have a bearing on the use of a stockpile if used on a “first come, first served” basis. Further research is required to optimize distribution and behaviour during a pandemic to ensure the cost-effectiveness of the stockpiling.

## Conclusions

Taking into account the existing evidence on pandemic influenza and the effectiveness of NAIs the decision should be to stockpile, provided a utilitarian decision-making framework is used of minimising expected losses and hence maximising expected benefits.

## Data availability

The data referenced by this article are under copyright with the following copyright statement: Copyright: © 2017 Watson SI et al.

Data associated with the article are available under the terms of the Creative Commons Zero "No rights reserved" data waiver (CC0 1.0 Public domain dedication). 

The data used to estimate the parameters in the model were obtained from documents compiled to assess pandemic influenza and thus represent the decision maker’s prior knowledge ^1^.

F1000Research: Dataset 1. Raw data of ‘stockpiling neuraminidase inhibitors for pandemic influenza usage’, 10.5256/f1000research.9414.d132653 ^44^.
