# Supplementary material for: Evidence synthesis and decision modelling to support complex decisions: stockpiling neuraminidase inhibitors for pandemic influenza usage
Source: F1000Res. 2017 Mar 16;5:2293. Originally published 2016 Sep 12. [Version 2] doi: 10.12688/f1000research.9414.2 (PMC5365214; doi:10.12688/f1000research.9414.2)
Supplement: Supplementary file 2 [file f1000research-5-12012-s0001.tgz › 82f995c3-308c-401d-a812-c3c3adfbbac6.docx]

**Appendix A: Methods**

The government is faced with the decision of whether to stockpile Tamiflu or not. We utilise a simple decision theoretic model based on a linear loss function to move from statistical results and prior knowledge to a decision. The objective in Bayesian decision problems is to choose the action that minimises the expected value of a loss function with respect to the posterior distributions, if data are available, or with respect to prior distributions, if no data are available, for uncertain parameters. In our case we have two actions, $a_{1}$ and $a_{2}$, corresponding to not stockpiling and stockpiling, respectively, and we have a vector of uncertain parameters, $\pi$, which includes the effectiveness of NAIs, $\theta$, the clinical attack rate of influenza, $CAR$, the case fatality ratio of pandemic influenza, $CFR$, the QALY losses associated with a pandemic influenza death, $QALY$, the probability of a pandemic occurring in the shelf life of NAIs, $Prob$, and the probability that a pandemic influenza death occurred in a person who was hospitalised. We specify these parameters later. The setting for our decision is an influenza pandemic and we will choose to stockpile NAIs if the expected losses of stockpiling are less than the expected losses for not stockpiling. We consider only deaths arising from stockpiling and no other potential outcome such as pneumonia.

The losses from not stockpiling are the expected value of the deaths that may occur due to pandemic influenza, $d_{lost}$. The losses from stockpiling are $d_{lost}$ plus the costs of stockpiling, $c$, minus the value of the deaths averted due to stockpiling $d_{averted}$. We specify a simple linear loss function: $L\left( a_{1},\pi\right)=d_{lost}$ and $L\left( a_{2},\pi\right)=d_{lost}+c-d_{averted}$. The Bayesian decision is to stockpile if $\rho\left( a_{2} \right)=E\left( L\left( a_{2},\pi\right) \right)<\rho\left( a_{1} \right)=E\left( L\left( a_{1},\pi\right) \right)$ which is equivalent to the decision rule to stockpile if $E\left( c-d_{averted} \right)<0$. Or, equivalently, the decision is to stockpile if the expected net benefits $E\left( d_{averted}-c \right)$ are positive.

We specify

$$d_{averted}=\lambda\times Prob\times Pop\times Treated\times CAR\times CFR\times QALY\times Hospital\times\left( 1-\theta\right)$$

where $c$ is the costs of stockpiling, $\lambda$ is the social willingness to pay for a quality adjusted life year (QALY), and $Treated$ is the proportion of symptomatic cases that receive NAIs.

The expected net benefits are

$$E\left( d_{averted}-c \right)=\lambda\times Pop\times Treated\times Hospital\times E\left( QALY \right)\times E\left( Prob \right)\times E\left( CAR\times CFR \right)\times\left( 1-E\left( \theta\right) \right)-c$$

where the expectations are taken with respect to the posterior distributions of each variable, and where we have assumed that $c$, $\lambda$, $Pop$, $Treated$, and $Hospital$ are known constants and that $\left( CAR\times CFR \right)$ and $\theta$ are independent. This specification can be seen as analogous to an evaluation of the cost-effectiveness of NAIs where it is stockpiled if its cost-effectiveness is less than $\lambda$.

**Parameters**

***Number of Pandemics***

The occurrence of a pandemic in the shelf life of NAIs is modelled as a Bernouilli distribution with probability $p$. The time period considered is ten years, the shelf life of NAIs. A uniform (0,1) distribution for $p$ was specified. For the data, each decade between 1900 and 2010 was considered an observation equal to one if a pandemic occurred and zero otherwise. We specified the following model for each decade $t=1,\ldots,11$:

$$x_{t}\sim Bern\left( p \right)$$

$$p\sim Unif\left( 0,1 \right)$$

***Clinical Attack Rate and Case Fatality Ratio***

The clinical attach rate and case fatality ratios were assumed to be observations from Beta distributions with parameters $\alpha_{CAR},\beta_{CAR}$ and $\alpha_{CFR},\beta_{CFR}$, respectively. Uniform (0, 1000) (hyper)prior distributions were specified for each parameter. These were updated using data from previous pandemics.

***QALY Losses Associated with an Influenza Death***

As above, the mean age of a pandemic influenza associated death was modelled as a Beta distribution, but scaled to the interval [0, 81.5], the upper limit representing the life expectancy at birth in the United Kingdom. Uniform (0, 1000) (hyper)prior distributions were specified for each parameter and updated with data from previous pandemics.

To calculate QALYs lost due a death, the estimated mean age at death was subtracted from 81.5. The net present value (NPV) of the lost years was calculated using a discount rate of 3.5%, and each year weighted by 0.8. Setting the upper limit at 81.5 may represent an underestimate as the life expectancy of a person dying in middle age is greater than 81.5.

***Effectiveness of Oseltamivir and Bias Correction***

The estimated effect size from the observational meta-analysis, $y$, is arguably biased with respect to the true effect size, $\theta$. We followed the bias modelling method outlined by Turner *et al* to ‘correct’ the estimated effect.^1^ We allowed for both proportional and additive biases, represented by $\beta$ and $\delta$, respectively, and assumed the model:

$$y\sim f\left( \beta\theta+\delta,s^{2} \right)$$

where $f\left( \mu,\sigma^{2} \right)$ is a general distribution with mean $\mu$ and variance $\sigma^{2}$. The proportional and additive biases are distributed as $\beta\sim f\left( \mu_{\beta},\sigma_{\beta}^{2} \right)$ and $\delta\sim f\left( \mu_{\delta},\sigma_{\delta}^{2} \right)$.

There are assumed to be different sources of bias, such as selection bias and performance bias, which contribute to the overall bias. A questionnaire was provided to each independent reviewer regarding different sources of bias within the study, and each reviewer provider their beliefs about the bias. The full elicitation process is described by Turner *et al*, along with methods for determining $\mu_{\beta}$, $\sigma_{\beta}^{2}$, $\mu_{\delta}$, and $\sigma_{\delta}^{2}$.^1^ Results were pooled between reviewers by taking medians of the mean and standard errors.

The effectiveness parameter $\theta$ is a relative risk. We converted the odds ratios in the observational data to relative risks.^2^

The bias corrected estimator of the effectiveness of oseltamivir was then calculated as:

$$\hat{\theta}=\frac{y-\delta}{\beta}$$

With corresponding estimate of the standard error:

$$SE\left( \hat{\theta} \right)=s^{2}+\hat{\theta}^{2}\sigma_{\beta}^{2}+\sigma_{\delta}^{2}$$

We have focussed here on what are described as ‘internal’ biases and not ‘external’ biases. This is because the external biases mostly relate to events as yet unobserved which are incorporated in the model. Similarly, variable access to the treatment is accommodated in the model. Our approach is consistent with Turner et al. who advocate that only internal biases are considered in a systematic review/meta-analysis.^1^ Our study provides the modelling corollary of such a review.

**Statistical Code for WinBUGS**

The code provided here is for WinBUGS and R, which the analysis was initially run in. However, in later versions the model was run in Stan, for which the code is available as a file alongside the article.

require(R2OpenBUGS)

require(coda)

model<-function(){

#loss function

l2 <- c - lambda * pop * car.1 * cfr.1 * age.4 * (1 - theta) * prob * p_hosp

#priors for parameters

prob ~ dbern(prob.1)

theta ~ dnorm(0.89,123.456)

car.1 ~ dbeta(alpha, beta)

cfr.1 ~ dbeta(alpha2, beta2)

age.1 ~ dbeta(r1,r2)

age.2 <- 82.5-age.1*81.5

age.3 <- round(age.2)

age.4 <- 0.8 * age.npv[age.3]

#model for clinical attack rate

for(i in 1:4){

car[i]~dbeta(alpha,beta)

}

alpha ~ dunif(0,1000)

beta ~ dunif(0,1000)

#model for case fatality ratio

for(i in 1:5){

cfr[i]~dbeta(alpha2,beta2)

}

alpha2 ~ dunif(0,1000)

beta2 ~ dunif(0,1000)

#model for remaining QALYs

for(i in 1:4){

age[i]~dbeta(r1,r2)

}

r1 ~ dunif(0,1000)

r2 ~ dunif(0,1000)

#model for probability of pandemic in a decade

for(i in 1:11){

freq[i]~dbern(prob.1)

}

prob.1~dunif(0,1)

#model for proportion of deaths hospitalised

tot_hosp ~ dbin(p_hosp,n_hosp)

p_hosp ~ dbeta(0.0001,0.0001)

}

model.file <- file.path(tempdir(),"model.txt")

write.model(model, model.file)

#data

params<-c("theta","l2","car.1","cfr.1","age.4","prob.1","p_hosp")

c <- 560000000

pop <- 50500000

obs<- 0.75

lambda<-20000

qaly<-18.3

car<-c(0.25,0.30,0.35,0.07)

cfr<-c(0.0028,0.025,0.004,0.004,0.0005)

age<-c(27,65,62,45)

age<-age/81.5

age.npv<-sapply(1:200,function(x)npv(x,0.035))

freq<-c(0,1,0,0,0,1,1,0,0,0,1)

tot_hosp<-125

n_hosp<-136

#prob<-1-(dpois(0,lambda=0.04)^10)

data<-list("c","pop","lambda","car","cfr","age.npv","age","freq","tot_hosp","n_hosp")

inits <- function() { list(alpha=1, beta =1,alpha2=1, beta2 =1, r1=1, r2=1, rate=0.3,p_hosp=0.5) }

out <- bugs(data, inits, params,model,codaPkg=TRUE,

n.iter=10000,n.thin=10,bugs.seed=5)

npv<-function(t,r){

npv<-0

for(i in 0:t){

npv<-npv+1/(1+r)^i

}

return(npv)

}

**References**

1 Turner RM, Spiegelhalter DJ, Smith GCS, Thompson SG*.* Bias modelling in evidence synthesis. *J R Stat Soc Ser A Stat Soc* 2009;**172**:21–47. doi:10.1111/j.1467-985X.2008.00547.x

2 Grant RL. Converting an odds ratio to a range of plausible relative risks for better communication of research findings. *BMJ* 2014;**348**:f7450–f7450. doi:10.1136/bmj.f7450

**Appendix B: Data Sources and Additional Results**

**Previous pandemics**

Table B1 shows clinical attack rates (CAR) and case fatality ratios (CFR) from previous influenza pandemics for which these data were available that were used in our model.^1^

Figure B1 shows the prior distributions for the CAR and CFR used in the model. Beta distributions were used and parameters were estimated from the data in table B1. Methods are presented in Appendix A.

**Oseltamivir Effectiveness and Bias Correction**

The adjusted effectiveness estimate used in this study was an odds ratio (OR) of 0.81 (95% confidence interval: 0.70, 0.93) as reported in Muthuri *et al* for the odds of mortality with neuraminidase inhibitors irrespective of timing compared to no treatment. To represent the reduction in the risk of mortality, we require a relative risk for the model. The baseline risk of mortality in the quoted study is approximately 10%. We convert to a RR using the formula:$RR=OR/(1-p+\left( p\times OR \right))$.^2^ This gives an estimated adjusted RR of 0.83 (0.71, 0.94).

Figure B2 shows the estimates for total additive biases estimated by each reviewer presented as the effect the reviewer would expect to observe if there was no intervention effect in the study. The bias corrected relative risk was 0.89 (0.71, 1.07).

**Table B1**. Previous pandemics and associated clinical attack rates and case fatality ratios. Data from UK government document on the mitigation of pandemic influenza.[2]

| **Year of Outbreak** | **Pandemic** | **Geographical Spread** | **Clinical attack rates (%)** | **Case fatality ratios (%)** | **Average age of case fatality** |
| --- | --- | --- | --- | --- | --- |
| 1889-92 | Asiatic or Russian Flu | Russia -> ROW | 60^a^ | 0.28 | - |
| 1918 | Spanish flu | Unknown | 25 | 2.5 | 27 |
| 1957 | Asian flu | China -> ROW | 30 | 0.4 | 65 |
| 1968 | Hong Kong flu | China -> ROW | 35 | 0.4 | 62 |
| 2009 | 2009 flu pandemic | Mexico/USA -> ROW | 7 | 0.05 | 45 |

^a^This figure was not used in the modelling since the UK government believes a CAR of 50% to be the ‘worst case scenario’.

ROW = Rest of world

**Figure B1.** Estimated densities for clinical attack rate (top left), case fatality ratio (top right), QALY losses associated with a pandemic influenza death (bottom left), and the probability of a pandemic occurring in a decade (bottom right).


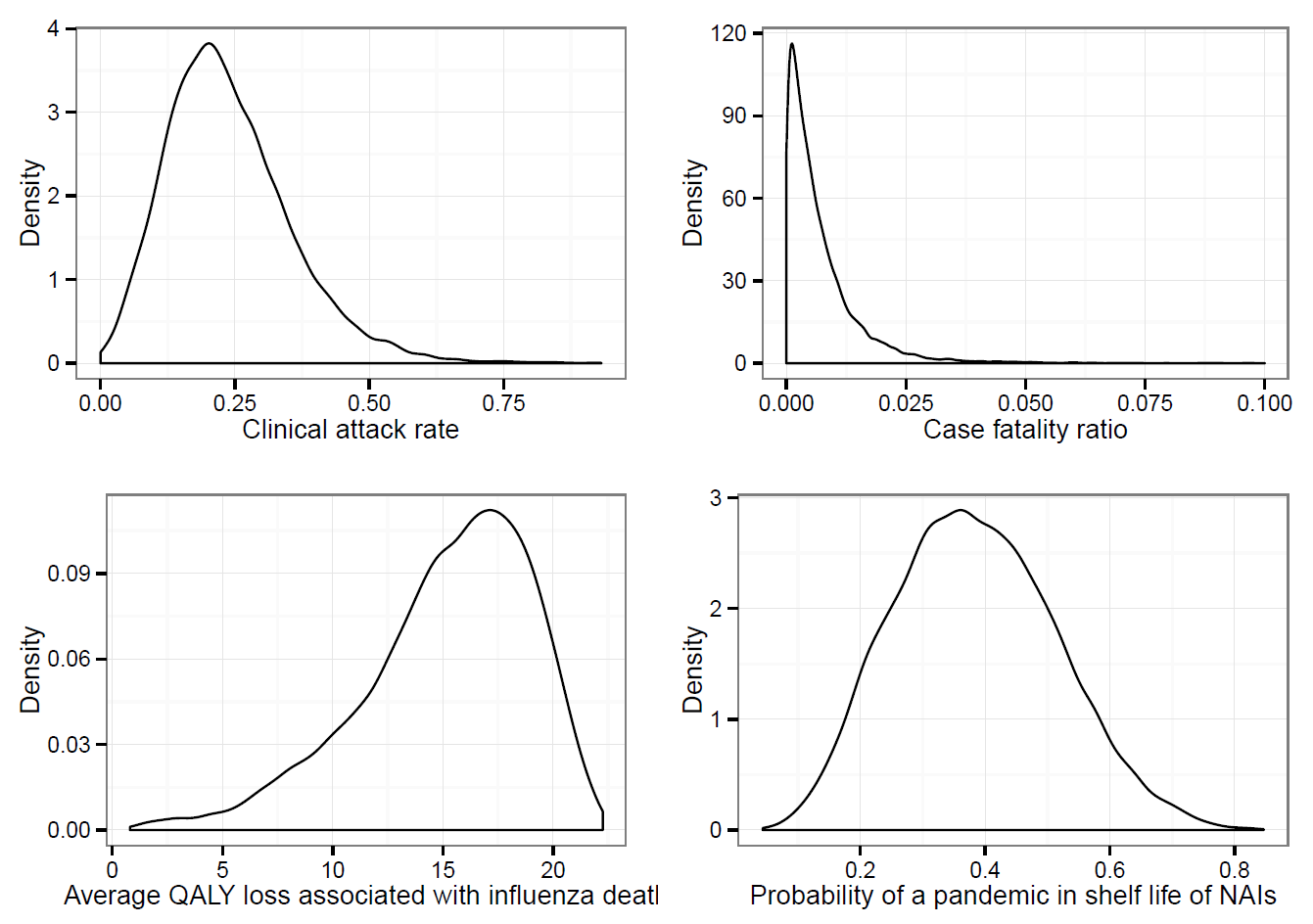


**Figure B2.** Estimated additive bias from five independent reviewers. Biases were assessed across a range of domains. This plot shows total additive biases which can be interpreted as the effect the reviewer would expect to observe if there was no intervention effect in the study.


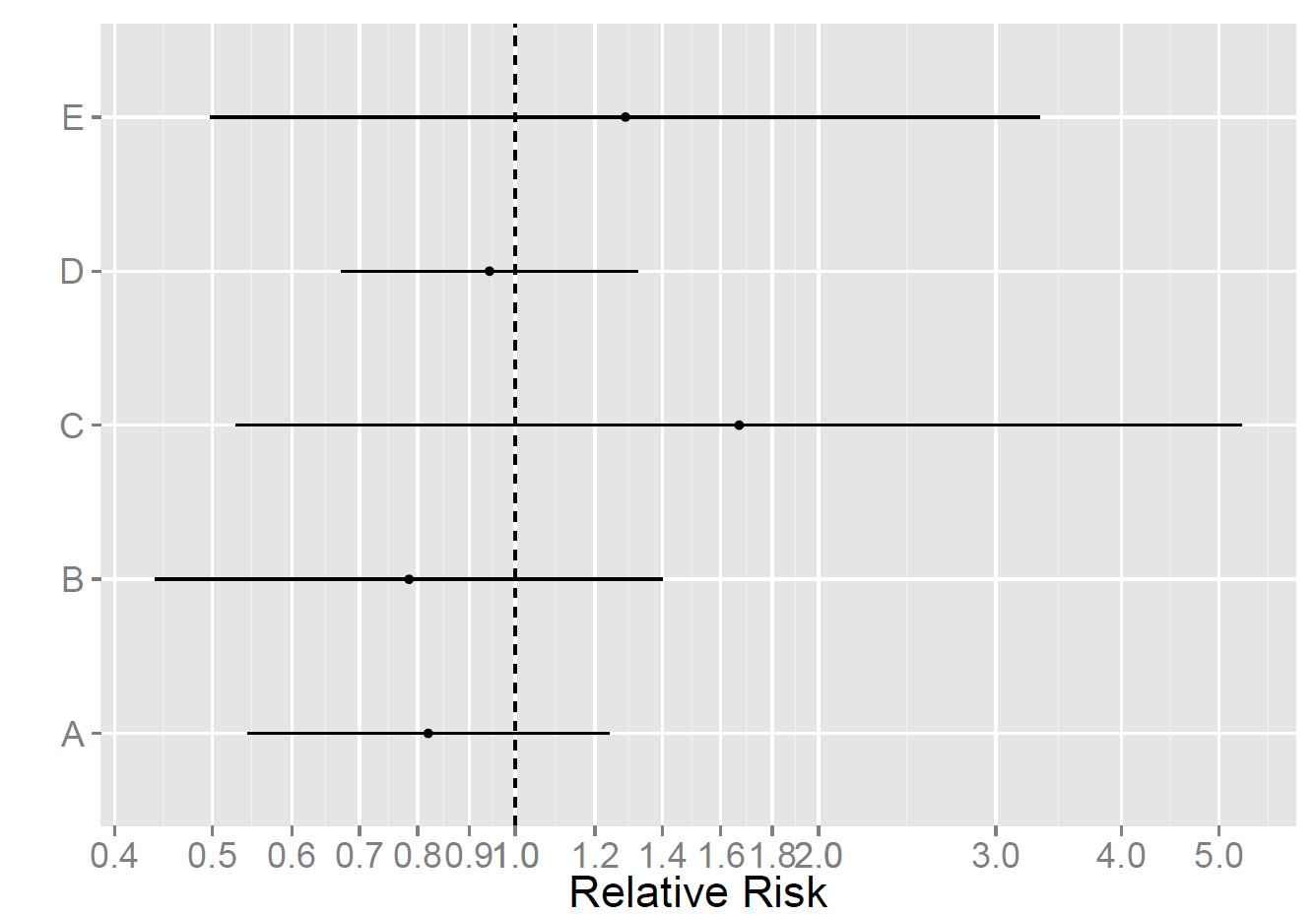


**References**

1. Department of Health. Scientific Summary of Pandemic Influenza & its Mitigation. London: 2011. https://www.gov.uk/government/uploads/system/uploads/attachment_data/file/215666/dh_125333.pdf. Date accessed: 24 September 2015
2. Grant RL. Converting an odds ratio to a range of plausible relative risks for better communication of research findings. Bmj 2014;**348**:f7450–f7450.
