# Supplementary material for: Evidence synthesis and decision modelling to support complex decisions: stockpiling neuraminidase inhibitors for pandemic influenza usage
Source: F1000Res. 2017 Mar 16;5:2293. Originally published 2016 Sep 12. [Version 2] doi: 10.12688/f1000research.9414.2 (PMC5365214; doi:10.12688/f1000research.9414.2)
Supplement: Peer review history

# Peer review: Referee response for version 2

**DOI:** 10.5256/f1000research.12012.r21030

**Published:** 2017-03-17

**Authors:** Joel Kelso

**Affiliations:** 1 School of Computer Science and Software Engineering, The University of Western Australia, Crawley, WA, Australia

This revised version (2) of the paper addresses all the criticisms I raised in my report.

I have read this submission. I believe that I have an appropriate level of expertise to confirm that it is of an acceptable scientific standard.

# Peer review: Referee response for version 1

**DOI:** 10.5256/f1000research.10138.r19365

**Published:** 2017-01-27

**Authors:** Joel Kelso

**Affiliations:** 1 School of Computer Science and Software Engineering, The University of Western Australia, Crawley, WA, Australia

This article reports on a probabilistic cost effectiveness of stockpiling neuraminidase inhibitor antiviral drugs to mitigate against pandemic influenza deaths. 

 The study is methodologically sound. The decision-theoretic approach which selects the optimal course of action based on the utility of each outcome and the probability of each outcome conditional on each decision is appropriate. In this case the actions are whether or not to stockpile NAIs for use in a pandemic, and the outcome is the cost of the antiviral stockpile and the expected number of deaths during the shelf-life period of the antivirals.

 The model of the expected number of pandemic deaths identifies is structurally sound and uses various appropriate data sources to quantify uncertainties present in all the parameters.

 The conclusion that stockpiling NAIs is cost-effective for a sufficiently high willingness-to-pay cost per QALY follows from the model and the data used.

 However, I think that more attention needs to be drawn to dependency of this result to the crucial antiviral effectiveness parameter. The methodology where all parameters are treated probabilistically in a uniform way is excellent; however additional one- or two- way sensitivity analyses are still valuable for providing insight into the effect of the most important parameters. The authors have done this for the proportion of hospitalised cases receiving antivirals; it seems appropriate to also do this for NAI effectiveness, given the ongoing debate on the subject.

 I have divided further comments into three sections: major essential revisions, commentary with discretionary additions, and minor technical clarifications needed.

 Essential Revision

 ------------------

1. As stated above, the reader would be well served with an additional figure similar to Figure 3, but plotting QALY threshold against mean NAI effectiveness. In my shallow experimentation with the author's model, it looks like at 20,000 GBP / QALY, NAIs cease being cost effective at around 0.94 effectiveness (relative risk, compared to 0.89).

Commentary

 ----------

 The suggestions made below I think might improve the article however I think the authors can best judge whether the additional effort and added complexity would be worthwhile or would be too diverting.

 I commend the authors for including the model code in the Appendix, I managed to run this code with relatively little effort.

1. The methodology of using expert opinion to mitigate potential bias in the studies estimating NAI effectiveness is a practical measure that is probably worthwhile. Some additional detail on the process would be appreciated. For example: how were assessors selected? How much time did the reviewers take in their bias estimates?
2. In the discussion it could be noted that in a future pandemic with a large CAR or CFR, the proportion of severe cases receiving hospital care and the level of care are likely to be lower, simple due to hospitals being overwhelmed. The estimates of proportion of deaths occurring in hospital are from the 2009 pandemic which was very mild.
3. If NAIs have any effect in preventing further transmission, e.g if they shorten the period of viral shedding, then mass administration of antivirals may reduce the overall attack rate and consequent mortality even if NAIs are not effective for mortality reduction of severe cases. As the study's model does not capture this, this is another way in which the study is conservative.

Minor Technical Revisions

 -------------------------

1. The CAR and CFR parameters used in the model are for a pandemic without NAI usage. Given that NAIs were used in the 2009 pandemic, should the CAR and CFR estimates for 2009 be included along side those of previous pandemics? If the 2009 CAR and CFR estimates are for example based on global data where NAI usage might be negligible that would be OK; but if they are based primarily on UK or US data they should possibly be excluded. 
2. In the Appendix page 4 there is a citation [20] that isn't given in a reference list.
3. In the last sentence of the 3rd paragraph, the RR derived based on the OR and 10% mortality is stated as 0.89. This is the same as the bias-corrected RR given in the next paragraph. Is this intentional? Or should it be the RR value based on the OR and 10% mortality (but without bias correction), in which case it should be 0.825 (from the formula).
4. The R / BUGS code in the Appendix worked almost without alteration. I found that I had to:
  Install BUGS (OpenBGUS).
  Hoist the npv function to the top.
  Remove the codaPkg=TRUE setting to obtain a result object.
  (also the "obs" and "qaly" values appear to be dead code)
  If F1000 allows additional appendix files this could be supplied as an additional plain ASCII file, to avoid scraping the text from the PDF and correcting resulting formatting.
5. I can't find the support for the n_hosp data value of 136. The tot_hosp value of 125 appears in the Donaldson BMJ paper. That paper gives 138 for the total number of confirmed deaths due to pandemic influenza.

I have read this submission. I believe that I have an appropriate level of expertise to confirm that it is of an acceptable scientific standard, however I have significant reservations, as outlined above.

# Response

**Published:** 2017-03-08

**Authors:** Sam Watson

**Affiliations:** University of Warwick, UK

We thank the review for their comments and detail our responses below, point by point. The referee's text is in Italics.

*Essential Revision*

*------------------*

1. *As stated above, the reader would be well served with an additional figure similar to Figure 3, but plotting QALY threshold against mean NAI effectiveness. In my shallow experimentation with the author's model, it looks like at 20,000 GBP / QALY, NAIs cease being cost effective at around 0.94 effectiveness (relative risk, compared to 0.89).*

We have replaced figure 3 to incorporate these considerations and additional commentary.

*Commentary*

*----------*

**

*The suggestions made below I think might improve the article however I think the authors can best judge whether the additional effort and added complexity would be worthwhile or would be too diverting.*

**

*I commend the authors for including the model code in the Appendix, I managed to run this code with relatively little effort.*

1. *The methodology of using expert opinion to mitigate potential bias in the studies estimating NAI effectiveness is a practical measure that is probably worthwhile. Some additional detail on the process would be appreciated. For example: how were assessors selected? How much time did the reviewers take in their bias estimates?*

We have added additional description  in the Methods section although we also refer the referee to the cited article

  

1. *In the discussion it could be noted that in a future pandemic with a large CAR or CFR, the proportion of severe cases receiving hospital care and the level of care are likely to be lower, simple due to hospitals being overwhelmed. The estimates of proportion of deaths occurring in hospital are from the 2009 pandemic which was very mild.*
2. *If NAIs have any effect in preventing further transmission, e.g if they shorten the period of viral shedding, then mass administration of antivirals may reduce the overall attack rate and consequent mortality even if NAIs are not effective for mortality reduction of severe cases. As the study's model does not capture this, this is another way in which the study is conservative.*

We will add these points to the discussion

*Minor Technical Revisions*

*-------------------------*

1. *The CAR and CFR parameters used in the model are for a pandemic without NAI usage. Given that NAIs were used in the 2009 pandemic, should the CAR and CFR estimates for 2009 be included along side those of previous pandemics? If the 2009 CAR and CFR estimates are for example based on global data where NAI usage might be negligible that would be OK; but if they are based primarily on UK or US data they should possibly be excluded. *

We would argue that the 2009 observed CAR and CFR are relevant data points to infer the distribution of possible CAR and CFR values. It is possible that mass NAI distribution may alter the parameters of these distributions, however, without further information it is not possible to model this. Excluding the 2009 pandemic would bias our estimates, and given the small amount of data, this data point provides a relatively large amount of information. We therefore opt to use all available data.

  

1. *In the Appendix page 4 there is a citation [20] that isn't given in a reference list.*

This has been amended.

  

1. *In the last sentence of the 3rd paragraph, the RR derived based on the OR and 10% mortality is stated as 0.89. This is the same as the bias-corrected RR given in the next paragraph. Is this intentional? Or should it be the RR value based on the OR and 10% mortality (but without bias correction), in which case it should be 0.825 (from the formula).*

We believe the referee may be in error, as it is 0.83 in the third paragraph of Appendix B. However, we have resubmitted the revised appendix to ensure the correct version is available.

  

1. *The R / BUGS code in the Appendix worked almost without alteration. I found that I had to:*
**
* Install BUGS (OpenBGUS).*
* Hoist the npv function to the top.*
* Remove the codaPkg=TRUE setting to obtain a result object.*
* (also the "obs" and "qaly" values appear to be dead code)*
**
* If F1000 allows additional appendix files this could be supplied as an additional plain ASCII file, to avoid scraping the text from the PDF and correcting resulting formatting.*

We have provided a file for use in the program Stan to run the program as well. The models were initially run in WinBUGS before ‘upgrading’ to Stan. We have noted this in the Appendix but opt to provide both pieces of code for people using both programs.

  

1. *I can't find the support for the n_hosp data value of 136. The tot_hosp value of 125 appears in the Donaldson BMJ paper. That paper gives 138 for the total number of confirmed deaths due to pandemic influenza.*

This typo has been amended.

# Peer review: Referee response for version 1

**DOI:** 10.5256/f1000research.10138.r17973

**Published:** 2017-01-18

**Authors:** Pasi M. Penttinen

**Affiliations:** 1 European Centre for Disease Prevention and Control, Stockholm, Sweden

This is a well designed, carefully executed and documented study, that provides important insights into the cost-effectiveness of national stockpiles of neuraminidase inhibitors to be used during influenza pandemics. 

 The analysis is relying on a number of key assumptions, such as the effectiveness of NAI antivirals against mortality due to influenza, the probability of a pandemic occurring during the shelf life of the stockpile and the proportion of pandemic influenza deaths occurring in hospital. Many of these assumptions are based on a limited or controversial evidence base, however the authors acknowledge and address most of these limitations.

 The assumption that most pandemic deaths occur in hospitals, is based on the observation during the 2009 pandemic in the UK, however in many countries, already during severe influenza A(H3N2) epidemics, and during many previous pandemics, the majority of deaths are likely to occur in the community, outside of hospitals. It is confusing that the authors compare the costs of a population wide (80%) stockpile with the estimated benefits on hospital mortality only. Although this is discussed in the second paragraph of discussion, it would be helpful to see an analysis or results taking also into account outpatient and community mortality.

 It is likely that such an analysis would be useful for other countries than UK. Please discuss briefly the limitations of this approach and these assumptions, when replicating the study in other settings (such as differences in societal willingness to pay per QALY).

 In Box 1. the two columns are not aligned when viewing as a pop-up on MS Internet Explorer.

 In Figure 1. the references to UK, and the national pandemic flu service are not helpful and distract from the more general main message of this figure.

I have read this submission. I believe that I have an appropriate level of expertise to confirm that it is of an acceptable scientific standard.

# Response

**Published:** 2017-03-08

**Authors:** Sam Watson

**Affiliations:** University of Warwick, UK

We thank the review for their comments and detail our responses below, point by point. The referee's text is in Italics.

*This is a well-designed, carefully executed and documented study, that provides important insights into the cost-effectiveness of national stockpiles of neuraminidase inhibitors to be used during influenza pandemics.*

**

*The analysis is relying on a number of key assumptions, such as the effectiveness of NAI antivirals against mortality due to influenza, the probability of a pandemic occurring during the shelf life of the stockpile and the proportion of pandemic influenza deaths occurring in hospital. Many of these assumptions are based on a limited or controversial evidence base, however the authors acknowledge and address most of these limitations.*

**

*The assumption that most pandemic deaths occur in hospitals, is based on the observation during the 2009 pandemic in the UK, however in many countries, already during severe influenza A(H3N2) epidemics, and during many previous pandemics, the majority of deaths are likely to occur in the community, outside of hospitals. It is confusing that the authors compare the costs of a population wide (80%) stockpile with the estimated benefits on hospital mortality only. Although this is discussed in the second paragraph of discussion, it would be helpful to see an analysis or results taking also into account outpatient and community mortality.*

 We did not consider non-hospital mortality as there were no data on the effectiveness of NAIs outside of the hospital setting, where there may be differences in compliance and other factors, when the study was conducted. We note that the way we have set up the analysis is to try to be as conservative as possible: the highest stated costs with a justifiable patient pool. On this basis we note that if a decision to stockpile is supported under our assumptions then it will certainly be supported if there is any benefit outside of the hospital. Recently published analyses outside of the hospital setting suggest a potential benefit ( https://doi.org/10.1093/cid/cix127), however we opt to remain conservative in our analyses.

*It is likely that such an analysis would be useful for other countries than UK. Please discuss briefly the limitations of this approach and these assumptions, when replicating the study in other settings (such as differences in societal willingness to pay per QALY).*

 We have amended the discussion to reflect this.

 In Box 1. the two columns are not aligned when viewing as a pop-up on MS Internet Explorer.

*This is an issue for the journal.*

 In Figure 1. the references to UK, and the national pandemic flu service are not helpful and distract from the more general main message of this figure.

*We have removed that box from the figure.*
